# Dimerization of Cdc13 is essential for dynamic DNA exchange on telomeric DNA

**DOI:** 10.1016/j.jbc.2025.110496

**Published:** 2025-07-17

**Authors:** David G. Nickens, Spencer J. Gray, Robert H. Simmons, Matthew L. Bochman

**Affiliations:** Molecular & Cellular Biochemistry Department, Indiana University, Bloomington, Indiana, USA

**Keywords:** DNA-binding protein, DNA–protein interaction, molecular biology, *Saccharomyces cerevisiae*, telomere, Cdc13, dynamic DNA exchange, OB fold

## Abstract

Eukaryotic single-stranded DNA (ssDNA)-binding proteins (ssBPs) protect telomeres from nuclease activity. In *Saccharomyces cerevisiae*, the ssBP Cdc13 is an essential protein that acts as a central regulator of telomere length homeostasis and chromosome end protection. Cdc13 has high binding affinity for telomeric ssDNA, with a very slow off-rate. Previously, we reported that despite this tight ssDNA binding, Cdc13 rapidly exchanges between bound and unbound telomeric ssDNA substrates, even at sub-stoichiometric concentrations of competitor ssDNA. This dynamic DNA exchange (DDE) is dependent on the presence and length of telomeric repeat sequence ssDNA and requires both Cdc13 DNA binding domains, OB1 and OB3. Here, we investigated if Cdc13 dimerization is important for DDE by characterizing the dimerization mutant Cdc13-L91R. Using mass photometry, we confirmed that Cdc13-L91R fails to dimerize in solution, even in the presence of ssDNA. DDE assays revealed that Cdc13-L91R fails to undergo ssDNA exchange compared to the recombinant wild-type protein. Biolayer interferometry demonstrated that this effect was not due to differences in ssDNA binding kinetics. Thus, dimerization of Cdc13 is essential for DDE, and we model how this may impact telomere biology *in vivo*.

Telomeres protect chromosome ends from fusion and recombination events (1, 2, 3, 4, 5). In *Saccharomyces cerevisiae*, an important component of end protection is the CST complex formed by Cdc13, Stn1, and Ten1 (5, 6, 7). Cdc13 is the primary ssDNA-binding protein (ssBP) of CST, and it binds with high affinity to G-rich DNA, giving it specificity for the telomeric TG_1-3_ repeat sequence (8, 9, 10). Cdc13 also plays important regulatory roles in telomere length homeostasis (11, 12, 13, 14). These include dissociation of the CST complex leading to telomerase activation, interaction with the catalytic subunit of Pol α-primase, and reassociation of CST following telomere elongation (15, 16, 17). Thus, Cdc13 tightly binds telomeric ssDNA and is a central regulator of telomere biology in budding yeast (8, 18, 19).

Cdc13 binds at or near 3′ telomeric ssDNA to protect against nuclease activity (18, 20, 21, 22). During G1 phase, the CST complex is bound to short (12–18 nt) 3′ telomere overhangs (17). As S phase begins, CST dissociates, leaving Cdc13 ssDNA bound (15, 23, 24). Cdc13 then interacts with the Est1 subunit of telomerase, activating the Est2 reverse transcriptase for telomere extension (15, 25, 26). As telomere elongation proceeds, Cdc13 must relocate to the newly synthesized telomere 3′ end to reform the CST complex (16). Due to the tight binding of Cdc13 to telomeric ssDNA, a significant amount of energy would be required to dissociate Cdc13 from its pre-replication binding site (27). Complicating this is that unbound Cdc13 is rapidly exported from the nucleus (28). Therefore, how does Cdc13, a ssBP with high affinity for telomeric ssDNA, translocate on its substrate to complete replication and restore end protection?

Several groups have tested whether the Pif1 helicase can remove Cdc13 from telomeric ssDNA *in vitro* (9, 29, 30), but the results are mixed. It is hard to conclude such conflicting data, but Pif1 can remove Cdc13 from ssDNA under certain conditions. However, we recently found that Cdc13 itself rapidly exchanges between telomeric ssDNAs in a process that we termed dynamic DNA exchange (DDE) (9). DDE is faster than helicase eviction of Cdc13 from ssDNA (9, 30). It is temperature-dependent, occurring only at physiological temperatures. Both the length and sequence of the unbound secondary ssDNA affect DDE. The length of the pre-bound substrate only weakly correlates with DDE activity until it reaches 50 nt, which significantly reduces DDE. Further, DDE is only observed with telomeric ssDNA; poly(dT) substrates do not support it. Thus, DDE by Cdc13 also demonstrates physiological sequence specificity.

Cdc13 truncation mutants revealed the protein determinants of DDE. Recombinant OB3 domain alone and an N-terminal truncation mutant lacking the OB1 domain (Cdc13ΔOB1) tightly bind to telomeric ssDNA but lack DDE activity (Fig. S1*A*) (9). We concluded that DDE requires both the OB1 and OB3 domains, but because Cdc13 is homodimeric in solution (31), it is unclear whether DDE requires the OB1 and OB3 domains from each subunit or from a single monomer within the dimer. Here, we used mass photometry, DDE assays, and BLI to confirm that the Cdc13-L91R mutant is a ssDNA-binding-competent monomer in solution and compare its ability to undergo DDE to wild-type (WT) dimeric Cdc13. We also propose an updated model for how Cdc13 moves to the new telomere end following telomerase extension without dissociating from the ssDNA.

## Results

### The Cdc13-L91R mutation disrupts dimerization

Having characterized the DNA and Cdc13 domain requirements for DDE (9), we sought to determine if Cdc13 dimerization is necessary. Thus, we needed to generate a monomeric mutant. Cdc13 behaves as an obligate homodimer *in vivo* and *in vitro* (31). The OB1 domain mediates this dimerization (31, 32), and SEC-MALS analysis indicates that the L91R mutation disrupts dimerization. However, the L91R mutant still binds ssDNA with positive cooperativity (8), suggesting that ssDNA may induce dimerization.

**Figure 1:**
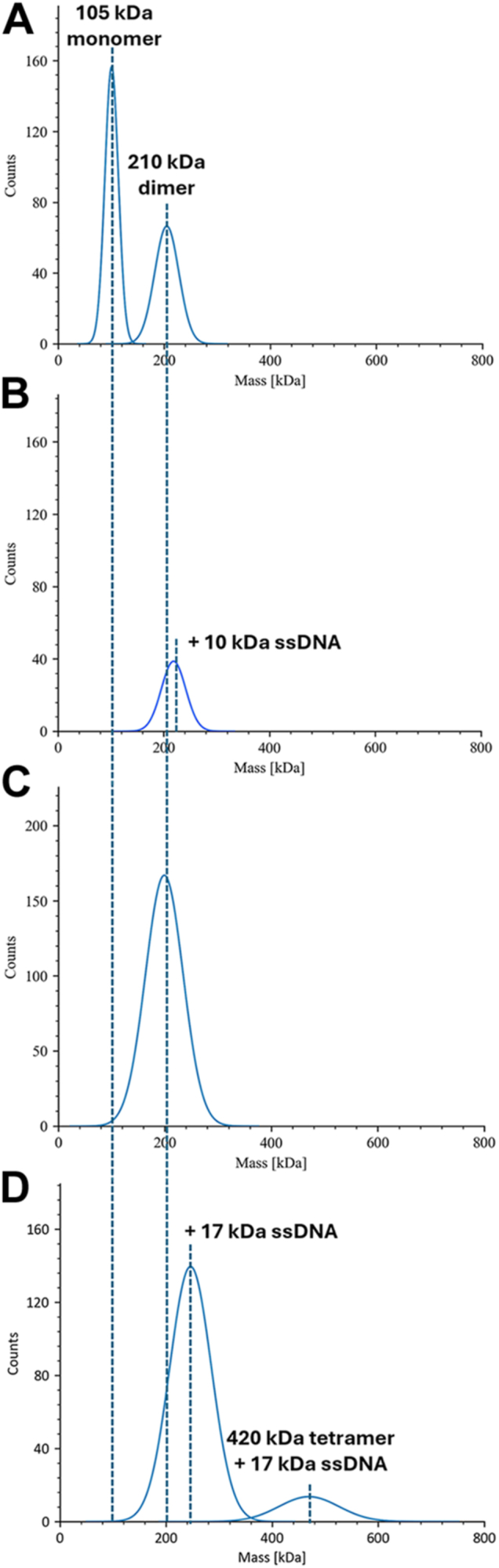
**Mass photometry of WT Cdc13**. *A*, recombinant Cdc13 exists as both monomers and dimers in PBS. *B*, pre-binding Cdc13 to Tel30G ssDNA yields only dimers. *C*, only Cdc13 dimers are observed in 1× binding buffer. *D*, the Tel50G substrate can be bound by two Cdc13 dimers. All assays contained 11.25 nM protein and 50 nM ssDNA, where indicated. The data shown are representative of ≥3 independent experiments.

To unambiguously determine if Cdc13-L91R is monomeric when bound to ssDNA, we generated a recombinant protein for mass photometry analysis. We typically dilute proteins with PBS for these assays, but found that PBS disrupts WT Cdc13 dimers, yielding a mixed population of monomers (∼105 kDa) and dimers (∼210 kDa) (Fig. 1*A*). Pre-binding Cdc13 to a 30-nt telomeric repeat sequence ssDNA substrate (Tel30G) prior to dilution yielded a single mass peak consistent with a Cdc13 dimer bound to an ∼10-kDa ssDNA (Fig. 1*B*) (33). The analysis does not preclude the possibility of two Cdc13 monomers independently binding the same substrate, but one would observe both one- and two-monomer binding events if that were true. When the mass photometry was repeated with the same buffer used in ssDNA binding assays, only Cdc13 dimers were observed (Fig. 1*C*). Similarly, in the binding buffer, both single- and double-dimer binding was observed upon the addition of an even longer 50-nt substrate (Tel50G; ∼17 kDa) that can accommodate two Cdc13 dimers (Fig. 1*D*). Thus, Cdc13-ssDNA binding buffer was used for all subsequent experiments.

**Figure 2:**
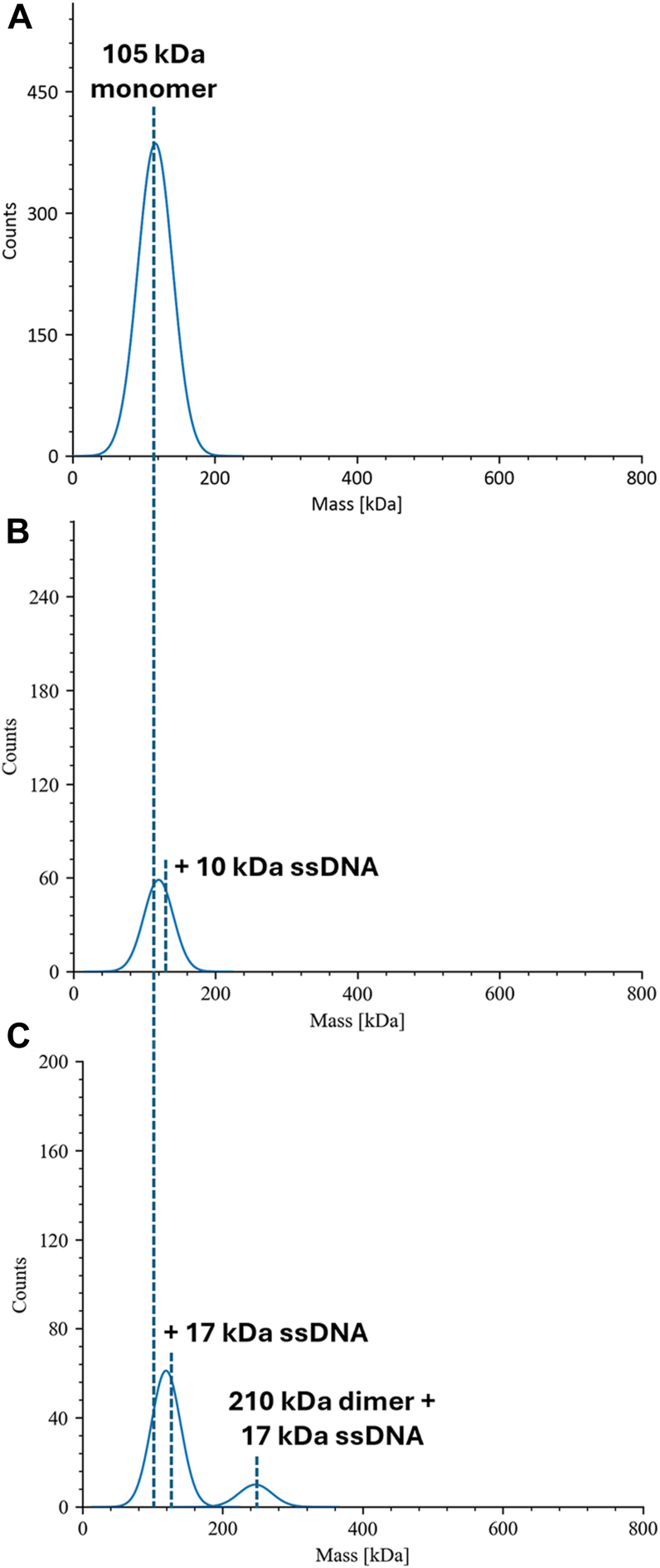
**Cdc13-L91R is monomeric is solution.***A*, recombinant Cdc13-L91R exists as a monomer in solution, and ssDNA binding (Tel30G) does not induce dimerization (*B*). *C*, Tel50G supports the binding of two Cdc13-L91R monomers. All assays were conducted as in Figure 1.

Analyzing recombinant Cdc13-L91R revealed that it only forms monomers in solution (Fig. 2*A*), consistent with the published SEC-MALS work (8). Binding to Tel30G also yielded a single mass peak consistent with one monomer bound to one ssDNA (Fig. 2*B*), confirming that DNA binding does not induce dimerization. Binding to the longer Tel50 did produce a small population of molecules with a mass of two Cdc13-L91R proteins (Fig. 2*C*), but we interpret these as independent protein molecules bound to the same substrate rather than dimers. In summary, our recombinant WT and L91R proteins exist as dimers and monomers, respectively, in the buffer conditions used to analyze DDE, so these reagents can conveniently be used to determine if DDE requires dimerization.

### Cdc13 dimerization is necessary for DDE

To assay for DDE, we typically use Tel30G conjugated to one of two different fluorophores: IR700 or IR800 (9). By prebinding Cdc13 to saturating amounts of IR800-Tel30G and then titrating in IR700-Tel30G, we can monitor the exchange of protein from one substrate to the other using gel shifts and two-color fluorescent imaging. We define DDE as competition for Cdc13 binding by sub-stoichiometric concentrations of secondary substrate. In contrast, a molar excess of secondary substrate is necessary for typical competition binding.

**Figure 3:**
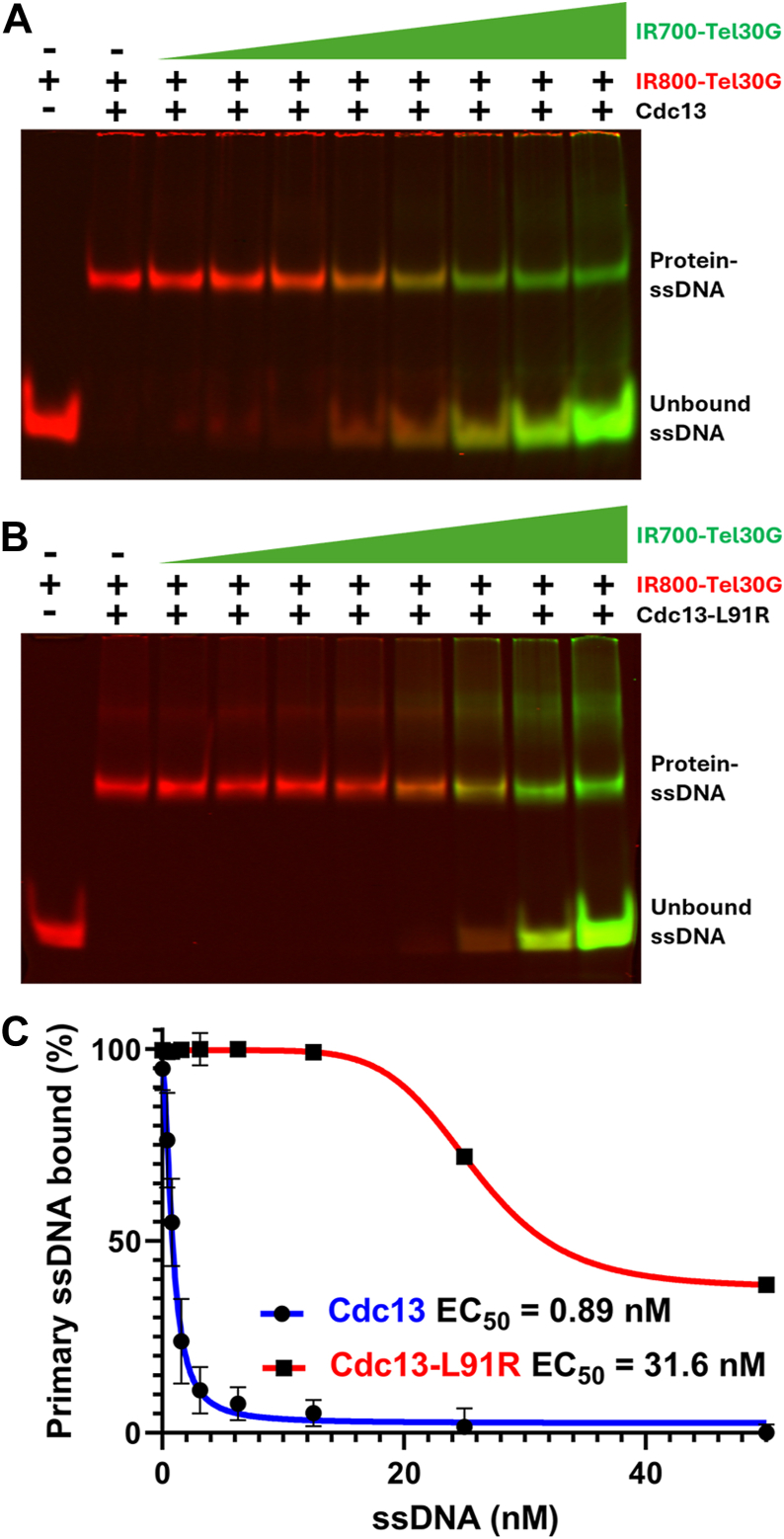
**Cdc13-L91R lacks DDE activity.***A*, representative gel of the dissociation of 3.75 nM WT Cdc13 dimer prebound to 2 nM IR800-Tel30G and exposed to increasing concentrations (0.2–15 nM) of IR700-Tel30G. *B*, representative gel of the dissociation of 3.75 nM Cdc13-L91R monomer prebound to 2 nM IR800-Tel30G and exposed to increasing concentrations of IR700-Tel30G. DNA migrating slower than the protein-ssDNA complex is also evident in this gel. Its identity is unknown but could be two monomers of Cdc13-L91R bound to a single substrate, or evidence that Tel30G forms intermolecular G-quadruplexes or other secondary structures. *C*, plot of the displacement of the initially bound IR800-Tel30G DNA by IR700-Tel30G. Error bars are the standard deviation of the mean of ≥3 replicates.

Figure 3*A* shows an example of WT Cdc13 undergoing DDE, where binding to IR800-Tel30G is disrupted by low concentrations of IR700-Tel30G. In contrast, much higher concentrations of IR700-Tel30G are required to compete for Cdc13-L91R binding (Figs. 3*B* and S2). These assays were performed in triplicate, and the results are quantified in Figure 3*C*. As reported (9), the EC_50_ of Cdc13 bound to 2 nM IR800-Tel30G and challenged with IR700-Tel30G is 0.89 nM, but that of Cdc13-L91R is 31.6 nM, a 35.5-fold difference. Because a stoichiometric excess of secondary substrate was needed to compete for Cdc13-L91R, we conclude that Cdc13-L91R is defective for DDE, and thus, DDE requires Cdc13 dimerization.

### Cdc13-L91R does not undergo DDE

An orthogonal method to observe DDE is BLI (9). Cdc13-ssDNA complexes are very stable, with a half-life of ∼2 days (27), while DDE occurs in seconds (9). Both of these phenomena can be observed for WT Cdc13 by BLI, where its off-rate from Tel30G ssDNA is minuscule over 24 h unless unlabeled Tel30G substrate is introduced to initiate DDE (9). Here, we used BLI to recapitulate these Cdc13-Tel30G binding results and to further characterize Cdc13-L91R (Fig. S3).

**Figure 4:**
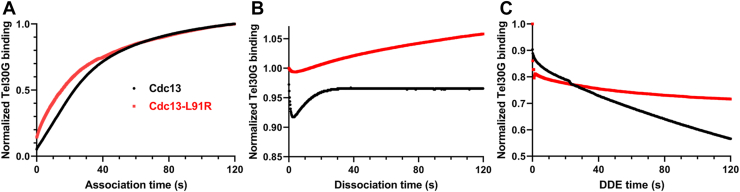
**BLI analysis of Cdc13 and Cdc13-L91R binding to telomeric ssDNA.***A*, association of 100 nM Cdc13 or Cdc13-L91R with immobilized Tel30G ssDNA was monitored for 2 min. *B*, Protein-ssDNA complexes were diluted into a large volume of DNA-free buffer, and dissociation was monitored for 2 min. *C*, the remaining protein-ssDNA complexes were exposed to free unlabeled Tel30G ssDNA in solution, driving either DDE (Cdc13) or simple binding competition (Cdc13-L91R). The curves shown are comprised of data points collected every 0.2 s and are representative of ≥3 independent experiments. The Cdc13 data are from (9) and were collected at the same time as the Cdc13-L91R data.

Both WT and Cdc13-L91R bind Tel30G with similar association kinetics (Fig. 4*A*), but they differ in the pre-DDE dissociation phase of the assay (Fig. 4*B*). As reported (9), full-length WT Cdc13 undergoes a brief initial dissociation and then reassociation with the ssDNA upon dilution of the protein–DNA complexes, but then no further dissociation is observed. Cdc13 truncation mutants that do not undergo DDE lack this phase in their BLI profiles (9), and Cdc13-L91R lacks it here (Fig. 4*B*). However, unlike truncation mutants, which all dissociate from the ssDNA (9), Cdc13-L91R continues to slowly associate with the Tel30G on the sensor. It is not until secondary substrate is flooded into the reaction during the DDE/competition step that Cdc13-L91R begins to dissociate from the ssDNA (Fig. 4*C*). It experiences a rapid initial drop in binding, followed by a slow and steady decrease. Although WT Cdc13 likewise undergoes a fast initial drop in binding, its subsequent dissociation is more rapid than Cdc13-L91R, consistent with the dimer undergoing DDE as opposed to simple binding competition displayed by the monomer.

## Discussion

### Models for DDE based on the Cdc13 dimer

Our *in vitro* assays only monitor intermolecular DDE between two ssDNAs, but we hypothesize that intramolecular DDE from one binding site to another along a single long telomeric ssDNA is possible. This is based in part upon similar phenomena displayed by ssBPs, such as the long-range lateral diffusion of RPA in single-molecule experiments (34). It is also based on the fact that Cdc13 must transition from the dsDNA-ssDNA junction at a short telomere toward the 3′-end of a growing telomere to support telomerase activity and fill-in by Pol α-primase (11, 12, 13, 14, 15, 16, 17). Crucially, there is no known mechanism for Cdc13 movement, and intramolecular DDE could explain it.

**Figure 5:**
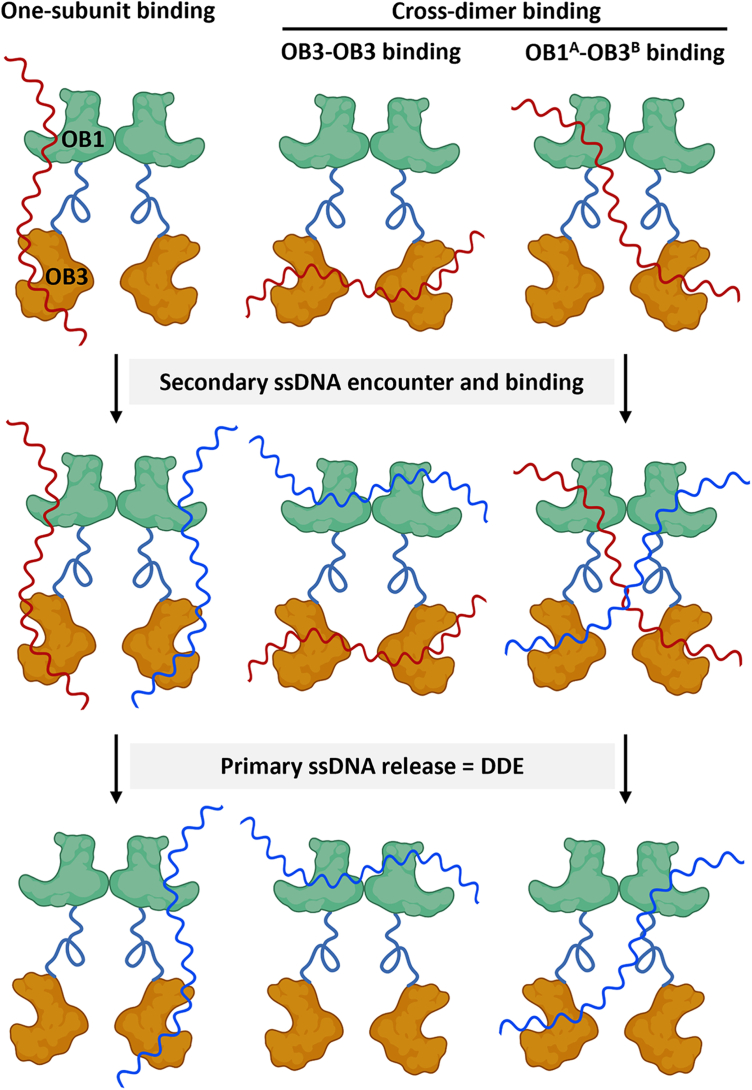
**Cdc13 dimer models for DDE.** Cdc13 homodimers contain four ssDNA binding sites: the OB1 and OB3 domains from each subunit. A long ssDNA substrate (*top, red*) can fill both OB1 and OB3 sites in one monomer (*left*), fill both high-affinity OB3 sites across the dimer (*middle*), or span the OB1 domain of subunit ‘A’ in the dimer and the OB3 domain of subunit ‘B’ (or *vice versa*). All three scenarios leave two open ssDNA binding sites that can be filled when encountering a secondary ssDNA substrate (*blue, middle*). Upon filling the second set of binding sites, the binding event is allosterically communicated across the dimer, leading to release of the primary ssDNA to complete the dynamic exchange of substrates (DDE, *bottom*). These models are depicted with two separate ssDNAs, but a single substrate of sufficient length, especially one that is actively being lengthened by telomerase, is predicted to fulfill the same role.

Our findings that Cdc13-L91R is monomeric (Fig. 2) and lacks DDE (Fig. 3) led us to propose that Cdc13 dimerization is critical for its movement along telomeric DNA. Given that there is one high- and one low-affinity ssDNA binding site per Cdc13 monomer, the L91R results imply that ssDNA binding across monomers or allosteric communication of the DNA-bound state of one monomer to another within the dimer is crucial for DDE (Fig. 5). Because Cdc13 binding to Tel50G inhibits DDE (9), long (≥50 nt) ssDNAs may be bound in a manner that fills all effective Cdc13 binding sites. This could mean filling both OB1 and OB3 sites in the dimer (4 sites filled) or both high-affinity sites (OB3s) and one low-affinity site (OB1 in subunit A or B). Binding to ssDNA <50 nt leaves some binding sites open for additional interaction and potential movement along telomeric DNA. The Cdc13-L91R mutant was previously characterized *in vivo*, where it results in shortened telomeres (32). One hypothesis to explain this is that failure of Cdc13 to dimerize could interfere with its Pol α-primase or Est1 interactions, which would lead to shortened telomeres (13, 31, 32).

### Cdc13-ssDNA complex structure prediction

To begin to understand how ssDNA length might regulate DDE, we modeled the Cdc13 structure with and without ssDNAs (Fig. S4). AlphaFold three predicts that the major Cdc13 dimer interface is the OB1 domain in each monomer (Fig. S4*A*), corresponding to published work (31, 32) and our Cdc13-L91R data (Fig. 2). In DNA-bound models, the dimer conformation changes as the length of bound ssDNA increases from 15 to 50 nt (Fig. S4, *B*–*D*). Conformational changes are also evident when Cdc13 is modeled bound to one telomere-like substrate (dsDNA with a 3′ ssDNA tail) *versus* two telomere substrates (Fig. S5). Notably, the Cdc13 dimer is predicted to synapse two telomeres (Fig. S5*B*), which would support the cross-dimer ssDNA binding model (Fig. 5).

Overall, we propose a refined model of Cdc13 telomere binding and translocation. During replication, the CST complex dissociates, allowing Cdc13 to interact with telomerase. During telomere extension, Cdc13 may dynamically exchange with newly synthesized ssDNA at a rate proportional its length. The movement is 3′-directed due to dsDNA blocking 5′ movement. Cdc13 translocation continues until the length of the G-strand reaches ≥50 nt, inhibiting DDE (9). As this occurs, the Cdc13 conformation changes to favor binding to other interaction partners such as Pol α. Because we demonstrated that DDE requires both OB1 and OB3 domains and dimers, Cdc13 may bind ssDNA with a single OB3 domain, both OB3s, or utilize a mixed OB1-OB3 binding site. When additional telomeric ssDNA has been synthesized of sufficient length, it can be bound by the open, unoccupied Cdc13 binding site(s), communicating the status of this secondary binding and releasing the initially bound ssDNA. The precise mechanism for DDE inhibition by long ssDNA is unknown, but it may involve filling of all Cdc13 ssDNA binding sites, stabilizing the protein-DNA interaction.

## Conclusions

George Box wrote that, “all models are wrong,” but that “The good scientist must have the flexibility and courage to seek out, recognize, and exploit such errors – especially his own” (35). In other words, all models are wrong, but some are useful. We hope that our models fall into the latter category, but they should also be interpreted with caution. For instance, the stoichiometry of the *S. cerevisiae* CST complex is unknown. Are two copies of Cdc13 present in CST such that the Cdc13 dimer is maintained? If so, can CST also undergo DDE, or does the interaction with Stn1 and Ten1 inhibit it? The analogous human complex (CTC1-STN1-TEN1) displays 1:1:1 stoichiometry in solution and itself oligomerizes (36), but CTC1 shares little homology with Cdc13. The more closely related CST complex from *Candida glabrata* displays a 2:4:2 or 2:6:2 stoichiometry between its Cdc13/Stn1/Ten1 subunits (37), but high-resolution analyses remain to be performed. Thus, answering our questions will require the generation of recombinant *S. cerevisiae* CST complexes that are stable in solution and competent for DDE analysis (9).

Similarly, our Figure 5 models invoke the use of all four Cdc13 dimer ssDNA binding sites, but the mutants tested to date impact both dimer subunits or yield monomers. Determining the mechanism of DDE will require the generation of mixed Cdc13 dimers containing one or more ssDNA-binding-deficient domains in the context of otherwise WT sites. Mixed Cdc13 dimers have been observed *in vivo* (31), so purification of differentially tagged Cdc13 and Cdc13 mutants could yield sufficient material to test our models.

In summary, we were the first to report DDE by Cdc13, and we demonstrate here that Cdc13 dimerization is necessary for this activity. The physiological implications of DDE have yet to be investigated, but we hope that the models, hypotheses, and questions highlighted above will guide further investigation of this phenomenon.

## Experimental procedures

### Reagents

All DNA substrates used in this work were purchased from Integrated DNA Technologies and included: IR700-Tel30G (/5IRD700/CGCCATGCTGATCCGTGTGGTGTGTGTGGG), IR800-Tel30G (/5IRD800/CGCCATGCTGATCCGTGTGGTGTGTGTGGG), biotinylated Tel30G (/5BiotiTEG/CGCCATGCTGATCCGTGTGGTGTGTGTGGG), and unlabeled Tel30G and Tel50G (GTGTGGGTGTGGTGTGGGTGTGGTGTGGGTGTGTGGGTGTGGTGTGGGTG). All other reagents were of molecular biology grade or higher and purchased from Sigma-Aldrich, unless otherwise noted.

### Biological resources

Vectors for over-expression of WT Cdc13 and Cdc13-L91R in *Spodoptera frugiperda* Sf9 tissue culture were provided by Dr Hengyao Niu. These pFastBac-based vectors encode a 5′-SUMO-His_6_ tag and a 3′-FLAG tag for purification. Cloning details are available upon request.

### Recombinant protein production

Sf9 cultures were grown in SF900 media and infected with baculovirus at a multiplicity of infection of 0.1. Cells were harvested by centrifugation after 3 days at 27 °C with gentle aeration. Pellets were stored at −80 °C. Frozen pellets were lysed in the presence of aprotinin, chymostatin, leupeptin, and pepstatin A (all at 5 μg/ml) and 1 mM phenyl-methyl-sulfonyl fluoride, as well as 10 μg/ml DNase I. Clarified lysates were purified as previously reported (9). Cdc13-L91R was purified identically to WT Cdc13. All protein preparations were assessed for DNA binding activity and contaminating nuclease activity using standard gel shift assays before experimental use. Representative images of the purified proteins are shown in Fig. S1*B*.

### Mass photometer analysis

Experiments were performed using a Two^MP^ mass photometer (Refeyn, Oxford, UK) to determine the oligomeric state of recombinant Cdc13 and Cdc13-L91R in solution. The protein mass standards BSA and thyroglobulin were used to generate a calibration curve for molecular weight determinations. All mass photometer experiments were performed in PBS or 1× DNA-binding buffer (25 mM HEPES (pH 8.0), 50 mM NaOAc, 150 mM NaCl, and 7.5 mM MgCl_2_) at room temperature. Proteins were diluted to 15 nM and incubated for 30 min at 30 °C in the presence or absence of 50 nM ssDNA substrate. The mass photometer was focused with buffer, and samples were diluted on slides to 11.25 nM protein. Samples were analyzed following the manufacturer’s protocols, and the data were graphed using DiscoverMP software.

### DDE assay

The DDE assays were performed as described (9). Briefly, IR800-labeled ssDNA substrate was mixed to a final concentration of 2 nM on ice with 3.75 nM Cdc13 (dimer concentration) or Cdc13-L91R (monomer concentration) in a final volume of 9 μl. Reactions were then incubated for 15 min at 30 °C before competitor IR700-labeled ssDNA was titrated in and incubated as before. Reaction products were separated on 8% native acrylamide gels and visualized using a LI-COR scanner.

### BLI

BLI experiments were performed to observe association, dissociation, and DDE using a BLItz instrument (FortéBio) as described (9). The data were plotted using GraphPad Prism software.

### Statistical analyses

Biological replicates (≥3) were performed for all assays. Unless otherwise noted, the plotted data points are the means of these replicates, and the error bars are the standard deviation. All graphs were plotted using GraphPad Prism software.

### Structure prediction

AlphaFold three models were rendered on the Google DeepMind AlphaFold Server (alphafoldserver.com) using default parameters. The protein sequence for *S. cerevisiae* Cdc13 from the S288c genetic background was obtained from the *Saccharomyces* Genome Database (yeastgenome.org). The sequences for telomeric ssDNA and a telomere-like substrate (*i.e.*, duplex DNA with a 3′ G-strand ssDNA tail) were input *in silico* with K^+^ ions. See Table S1 for additional details. Structures were visualized and analyzed in Chimera X (https://www.cgl.ucsf.edu/chimerax).

## Data availability

The data underlying this article are available in the article, in its supporting information, or will be shared upon reasonable request to the corresponding author.

## Supporting information

This article contains supporting information.

## Conflict of interest

The authors declare that they have no conflicts of interest with the contents of this article.
