# Supplementary material for: Dimerization of Cdc13 is essential for dynamic DNA exchange on telomeric DNA
Source: J Biol Chem. 2025 Jul 17;301(8):110496. doi: 10.1016/j.jbc.2025.110496 (PMC12362098; doi:10.1016/j.jbc.2025.110496)
Supplement: Supporting information [file mmc1.docx]

**SUPPORTING INFORMATION**

**Title:** Dimerization of Cdc13 is essential for dynamic DNA exchange on telomeric DNA

**Authors and Affiliations:**

David G. Nickens, Spencer J. Gray, Robert H. Simmons III, and Matthew L. Bochman*

Molecular & Cellular Biochemistry Department, Indiana University, Bloomington, IN 47405, USA

*To whom correspondence should be addressed. Tel: +1 812 856 2095; Email: [bochman@iu.edu](mailto:bochman@iu.edu)

**Supporting information included:**

**Supplementary Table S1.** AlphaFold 3 inputs.

**Supplementary Figures**

**Figure S1.** Domain schematic of *Saccharomyces cerevisiae* Cdc13.

**Figure S2.** Cdc13-L91R lacks DDE activity.

**Figure S3.** Example BLI assays to observe DDE.

**Figure S4.** AlphaFold modeling of apo- and ssDNA-bound Cdc13 dimers.

**Figure S5.** Predicted structures of Cdc13 homodimers bound to one and two telomeres.

**Supplementary Table**

**Table S1.** AlphaFold 3 inputs.

| **Protein, DNA, or ion** | **Sequence** |
| --- | --- |
| Cdc13 | MDTLEEPECPPHKNRIFVSSSKDFEGYPSKAIVPVQFVALLTSIHLTETKCLLGFSNFERRGDQSQEDQYLIKLKFKDRGSERLARITISLLCQYFDIELPDLDSDSGASPTVILRDIHLERLCFSSCKALYVSKHGNYTLFLEDIKPLDLVSVISTISTKSTNSSKHSSSELISECDLNNSLVDIFNNLIEMNRDEKNRFKFVKLIHYDIELKKFVQDQQKVLSQKSKAAAINPFFVPNRLGIPYIESQNEFNSQLMTLNVDEPTTDISNMGEEMHDSADPIEDSDSSTTSSTGKYFSSKSYIQSQTPERKTSVPNNWHDDDSGSKRKRKLSFHSPNASSIRKAISYEQLSLASVGSVERLEGKIVGMNPPQFASINEFKYCTLKLYFTQLLPNVPDKVLVPGVNCIEIVIPTRERICELFGVLNCQSDKISDILLLEKPDRISVEVERILWDNDKTASPGMAVWSLKNISTDTQAQAQVQVPAQSSASIDPSRTRMSKMARKDPTIEFCQLGLDTFETKYITMFGMLVSCSFDKPAFISFVFSDFTKNDIVQNYLYDRYLIDYENKLELNEGFKAIMYKNQFETFDSKLRKIFNNGLRDLQNGRDENLSQYGIVCKMNIKVKMYNGKLNAIVRECEPVPHSQISSIASPSQCEHLRLFYQRAFKRIGESAISRYFEEYRRFFPIHRNGSHLAKLRFDEVKHEPKKSPTTPALAEHIPDLNADVSSFDVKFTDISSLLDSSARLPRPQQTHKSNTLYSCEGRIIAIEYHASDLCFHITNELPLLQTRGLAPERVLQLHIITSKNFAYFFNRSSAYLQRQPLEEKYTQLAQFLGHSFKFNITSSLTLFPDTTVALQIWCPIECTFRELQQQLAHPKVAAAPDSGSLDCAINATVNPLRLLAAQNGVTVKKEEDNDDDAGAVPTS |
| OB1 domain | MDTLEEPECPPHKNRIFVSSSKDFEGYPSKAIVPVQFVALLTSIHLTETKCLLGFSNFERRGDQSQEDQYLIKLKFKDRGSERLARITISLLCQYFDIELPDLDSDSGASPTVILRDIHLERLCFSSCKALYVSKHGNYTLFLEDIKPLDLVSVISTISTKSTNSSKHSSSELISECDLNNSLVDIFNNLIEMNRDEKNRFKFVKLIHYDIELKKFVQDQQKVLSQKSKAAAINPFFVPN |
| OB3 domain | KMARKDPTIEFCQLGLDTFETKYITMFGMLVSCSFDKPAFISFVFSDFTKNDIVQNYLYDRYLIDYENKLELNEGFKAIMYKNQFETFDSKLRKIFNNGLRDLQNGRDENLSQYGIVCKMNIKVKMYNGKLNAIVRECEPVPHSQISSIASPSQCEHLRLFYQRAFKRIGESAISRYFEEYRRFFPIHRNGSHLAKLRFDEVKHEP |
| Tel15G | 5’-TGTGGTGTGTGTGGG-3’ |
| Tel30G | 5’-CGCCATGCTGATCCGTGTGGTGTGTGTGGG-3’ |
| Tel50G | 5’-GTGTGGGTGTGGTGTGGGTGTGGTGTGGGTGTGTGGGTGTGGTGTGGGTG-3’ |
| Telomere-like DNA | 5’-GGGTGTGTGGGTGGTGGGTGTGTGGGTGGTGGGTGTGGTGGTGTGGG-3’  5’-ACCACCCACACACCCACCACCCACACACCC-3’ |
| Ions | K^+^ |

**Supplementary Figures**

**
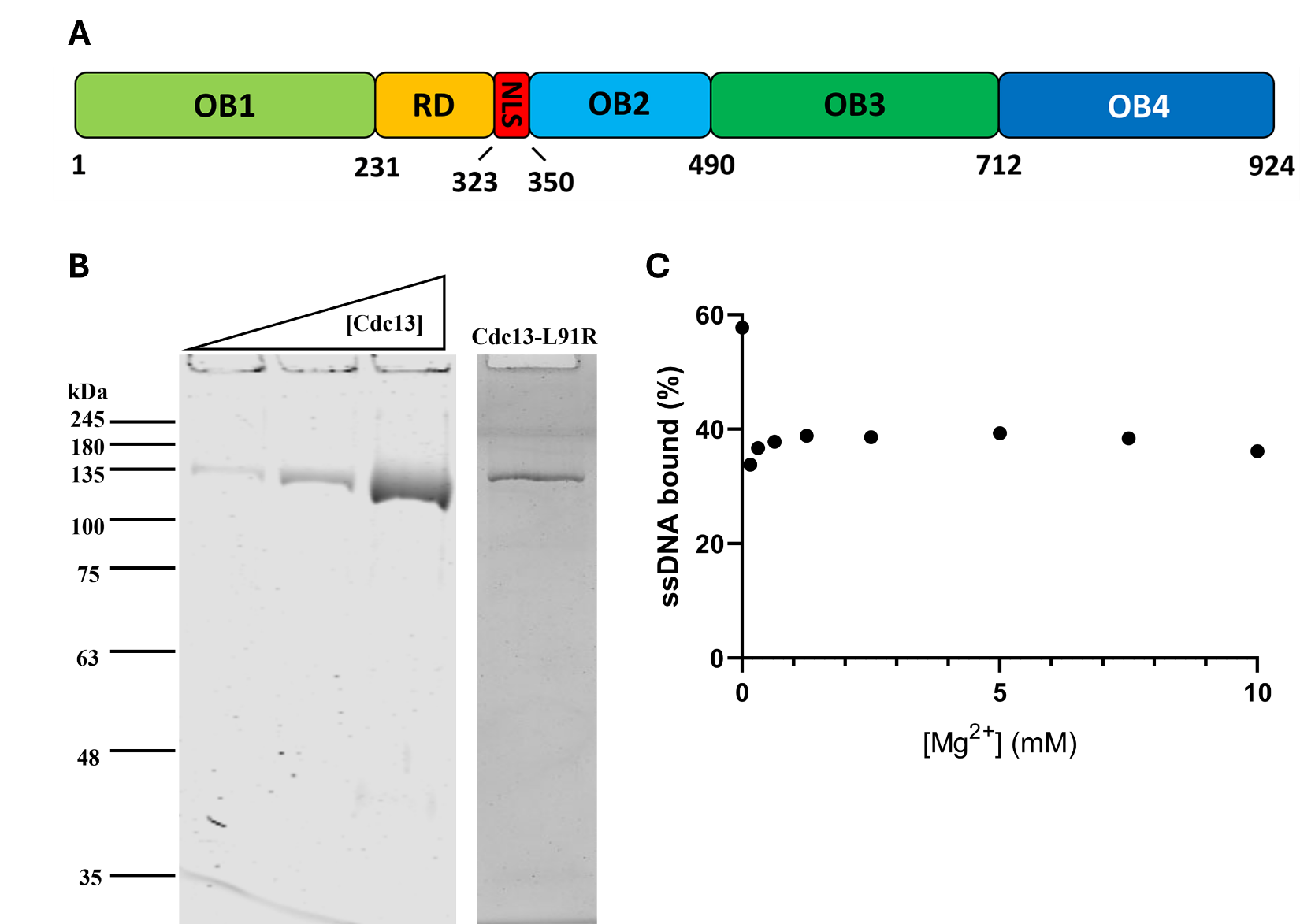
**

**Figure S1. Domain schematic of *Saccharomyces cerevisiae* Cdc13.** A) The domains comprising full-length Cdc13 are shown to scale, with the numbers below the image indicating the amino acid number at the domain boundary. RD, telomerase recruitment domain; NLS, nuclear localization sequence. B) Representative Coomassie-stained DSD-PAGE gel images of recombinant Cdc13 and Cdc13-L91R preparations. The Cdc13 titration on the left demonstrates the high purity of the proteins. C) Lack of Mg^2+^ in binding buffer inhibits DDE, but concentrations of Mg^2+^ in the range of 0.15-10 mM display similar stimulatory effects.

**
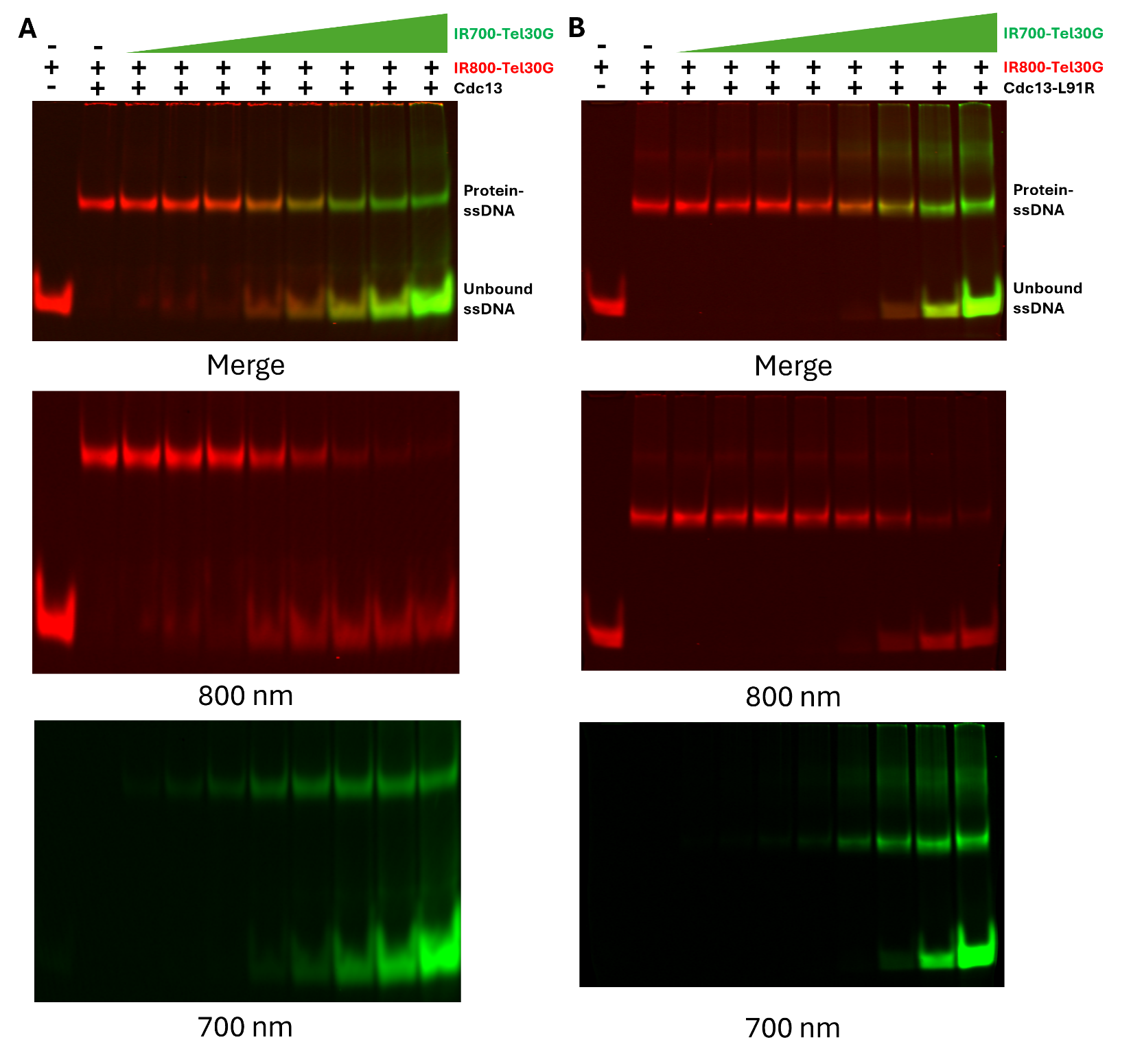
**

**Figure S2. Cdc13-L91R lacks DDE activity.** These data are related to Figure 3 from the main text. The separate 700- and 800-nm channels of the DDE assays are shown for both Cdc13 (A) and Cdc13-L91R (B).

**
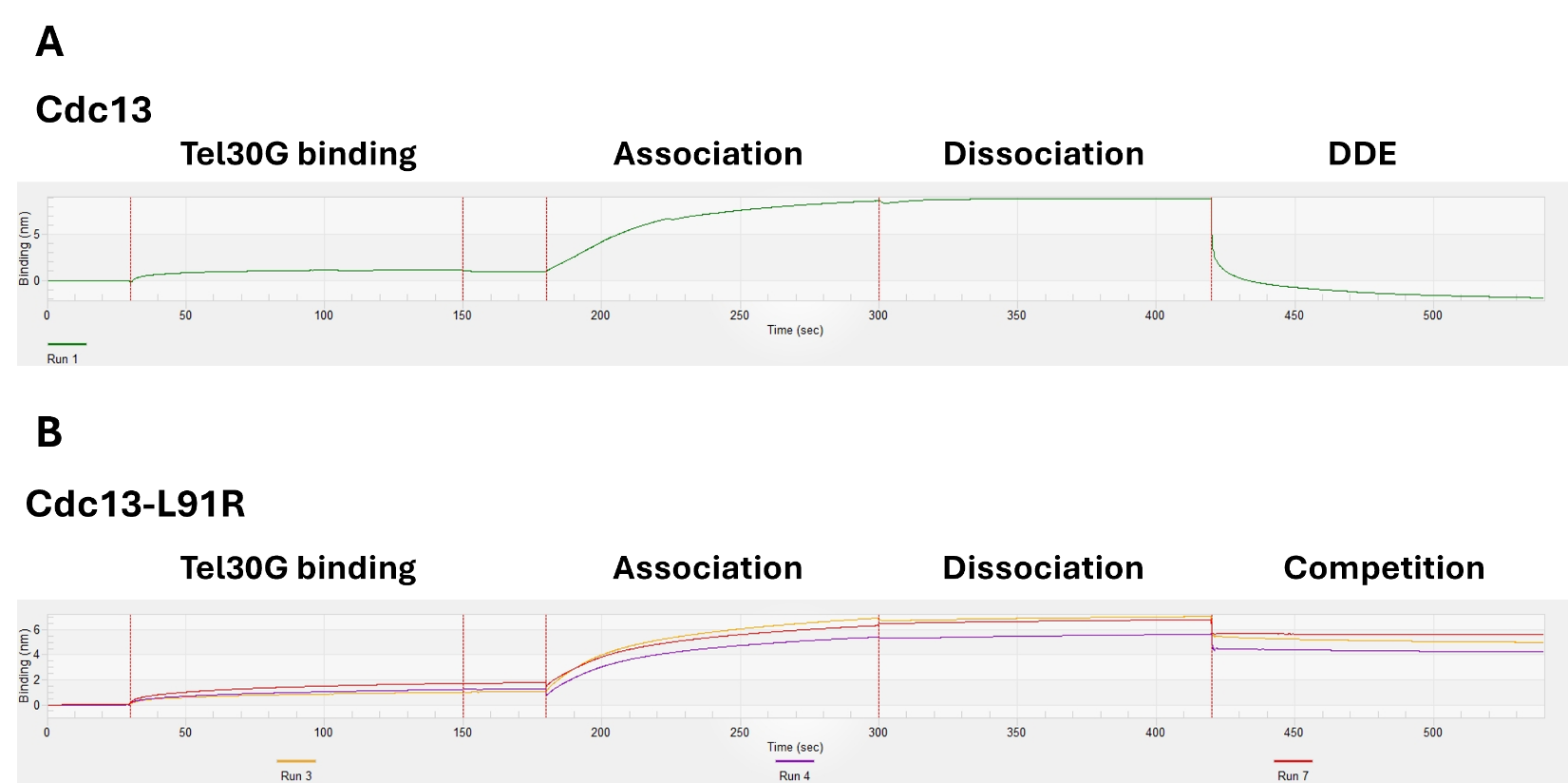
**

**Figure S3. Example BLI assays to observe DDE.** In a typical assay, biotinylated Tel30G ssDNA was immobilized on a streptavidin-coated BLI sensor (Tel30G binding), washed, exposed to recombinant protein for ssDNA binding (Association), exposed to a large volume of buffer to allow dissociation of the protein from the ssDNA (Dissociation), and then exposed to a large volume of buffer containing a molar excess of competitor ssDNA to allow DDE or binding competition to occur (DDE/Competition).


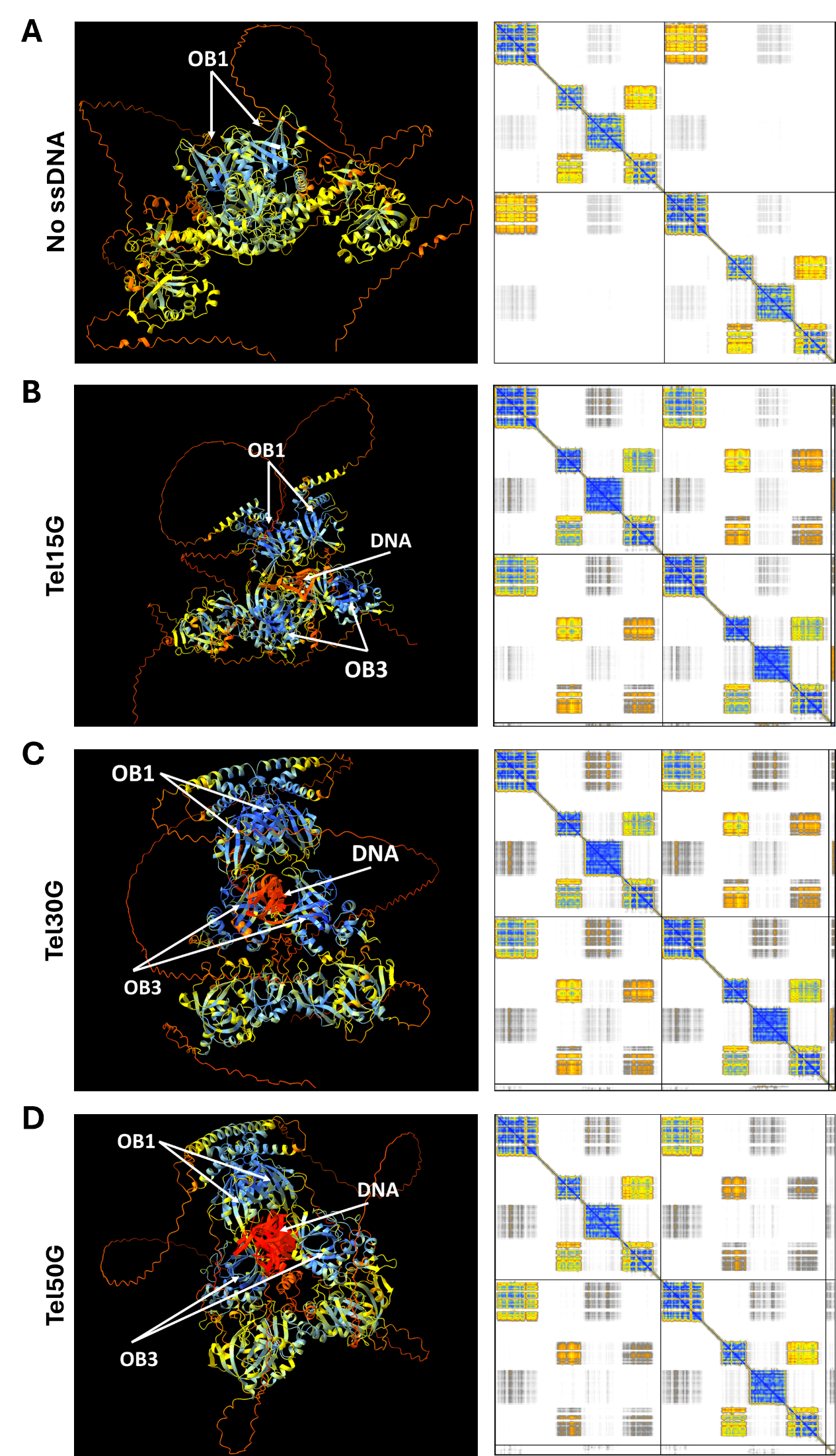


**Figure S4. AlphaFold modeling of apo- and ssDNA-bound Cdc13 dimers.** The AlphaFold 3 server was used to model the Cdc13 dimer (A) and the dimer bound to 15 (B), 30 (C), or 50 nt (D) of telomeric repeat sequence ssDNA. The positions of the OB1 and OB3 domains, as well as the ssDNA substrate, are labelled. Predicted alignment error plots are shown to the right of each structure. Intra-subunit contacts are shown in the top left and bottom right squares; inter-subunit contacts are shown in the others. Protein-ssDNA contacts are Cooler colors indicate higher confidence in the prediction.


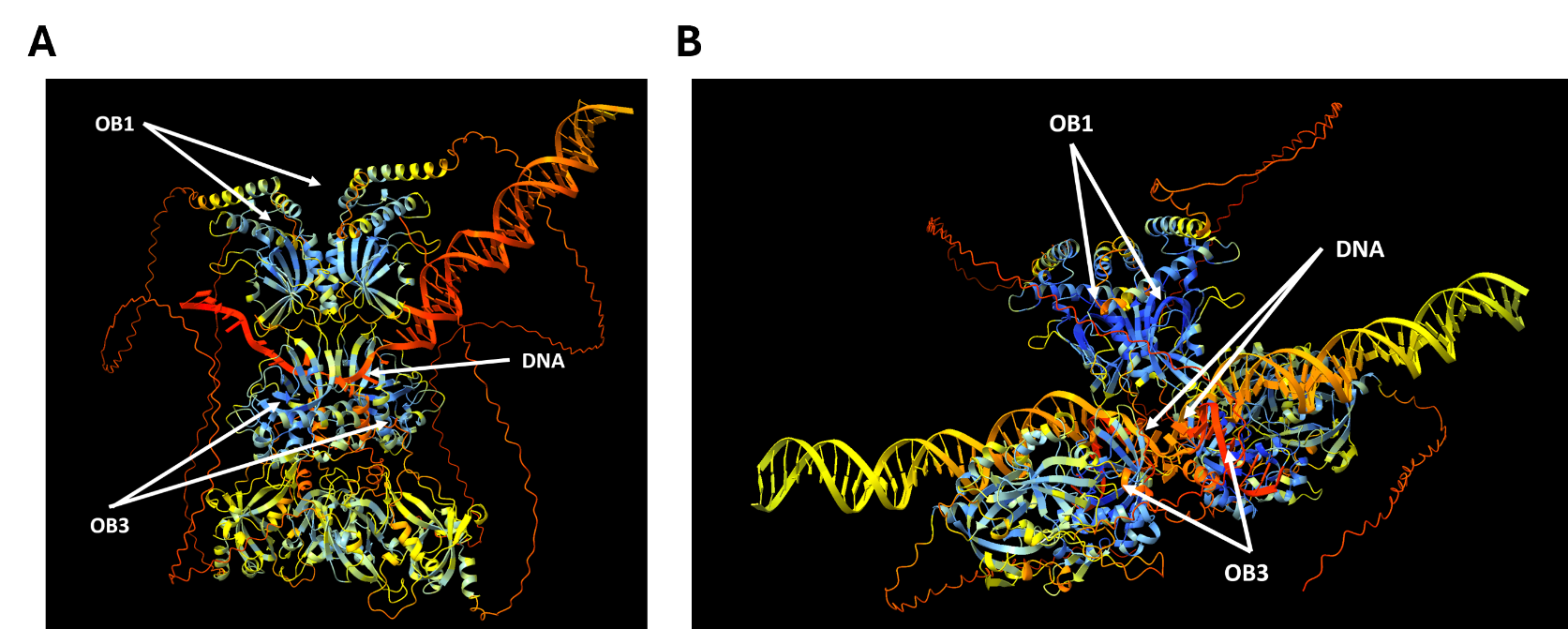


**Figure S5. Predicted structures of Cdc13 homodimers bound to one (A) and two (B) telomeres.** The telomere-bound Cdc13 dimer structures were predicted using the AlphaFold 3 server.
